# Model-based optimization of the primary drying phase of oral lyophilizates

**DOI:** 10.1016/j.ijpx.2020.100057

**Published:** 2020-10-31

**Authors:** Brecht Vanbillemont, Thomas De Beer

**Affiliations:** Laboratory of Pharmaceutical Process Analytical Technology (LPPAT), Department of Pharmaceutical Analysis, Faculty of Pharmaceutical Sciences, Ghent University, Ottergemsesteenweg 460, 9000 Ghent, Belgium

**Keywords:** Orodispersable tablets, Process optimization, Dynamic processing, Cold-form blisters, Freeze-drying

## Abstract

Oral lyophilizates also called orally disintegrating tablets (ODTs) are a patient friendly and convenient dosage form. They are manufactured by dosing a suspension in blister cups and subsequently freeze-drying these blisters to achieve porous tablets that disintegrate quickly (< 10 s) when placed upon the tongue. This paper proposes a mechanistic model of the primary drying phase of these oral lyophilizates processed in cold-form blisters. A heat transfer coefficient (*K*_*v*_) and dried layer resistance (*R*_*p*_) are regressed and applied in a dynamic optimization of the primary drying phase. The optimization exercise showed the possibility of ultra-short sublimation times for polyvinyl acetate (PVA) based formulations with a primary drying time of 3.68 h for a 500 mg acetaminophen tablet.

## Graphical abstract

**Figure:**
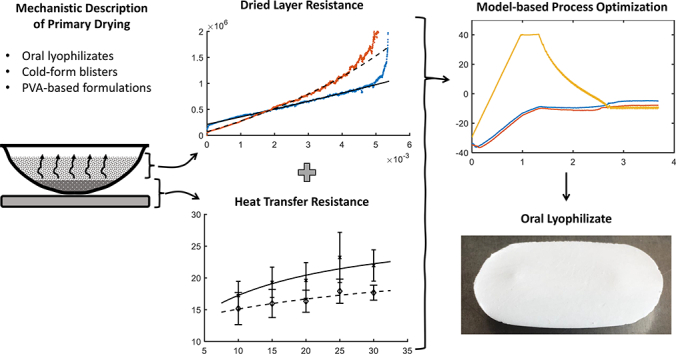

## 1 Introduction

The oral delivery route is the most popular method of drug administration. It is considered to be the most convenient, easy, and possesses the least amount of drug administration risks. Orally disintegrating tablets (ODTs) are unit doses that disintegrate quickly (<30 s according to the FDA) when orally administered (CDER, 2008). The tablet liquefies in the presence of saliva and the resulting liquid can subsequently be swallowed (CDER, 2008). Drug substances can either be absorbed via the buccal, the sublingual or the oral route. Some of these pre-gastric absorption routes can give clinical advantages as evidenced by the case of a selegiline ODT, which encountered reduced first pass effects (Clarke and Jankovic, 2006). Furthermore, administration of ODTs result in a higher therapy compliance compared to traditional tablets, especially in patient populations with dysphagia. (Sastry et al., 2000) There are also economic benefits as ODTs are generally developed as line-extensions which grant extended market exclusivity and patent protection. However, product- and production-wise it can be challenging to ensure an acceptable taste, an acceptable disintegration time, a sufficient mechanical strength, an adequate package design to safeguard the tablets, and a tolerable tablet size with sufficient dosing.

There are two main industrial ways of manufacturing ODTs. The foremost used technique of producing these tablets is by compaction of high water-soluble excipients (Badgujar and Mundada, 2011; Hirani et al., 2009; McLaughlin et al., 2009). The highly water-soluble excipients are needed to accelerate the disintegration time of these tablets since compression will lead to very low porosity's inhibiting the penetration of water. The disintegration time of compressed tablets can be reduced towards 30 s with the addition of significant amounts of superdisintegrants or effervescent components (Al-khattawi and Mohammed, 2013; Pahwa and Gupta, 2011). Another point of concern for compressed tablets is the dose accuracy in the case of low-dosed tablets. A direct compression tablet press is based on volumetric dye filling of the powder mixture containing the active pharmaceutical ingredient (API) and excipients. However, powders can have difficult flow properties or exhibit segregation of components which would lead to a sub-optimal dose accuracy. An excellent dose accuracy is at uttermost importance in low-dose medicines or drugs with a narrow therapeutic index. A solution exists by agglomeration of the powders to granules which can subsequently be compressed. However, not all forms of granulation are compatible with superdisintegrants or effervescent components as they need to stay dry during processing as their effect is based on expansion in contact with water. Moreover, direct compression and especially compression after granulation will lead to the disintegration of the tablet into a gritty mixture of powdered particles which does not lead to a pleasant mouthfeel. At last, due to the many powder streams leading to the compression process, many airborne particles are formed and in the case of toxic or highly therapeutic active components (i.e. corticosteroids or oncologicals) a complete isolation of the equipment is necessary to avoid cross-contamination or health risks towards the operators (Qiu et al., 2009).

Another method of producing ODTs is lyophilization where the active component is entrapped or dissolved in a highly porous matrix. The highly porous matrix will promote the penetration of water leading to superfast disintegration times as quick as 5 s. Moreover, the disintegration of the lyophilized ODT will result in a liquid with a nice mouthfeel. The API is typically dissolved in, or kept in suspension of, a liquid bulk formulation before lyophilization. Such a liquid formulation is much easier to dose volumetrically than powder mixtures, leading to generally higher dose accuracies compared to compressed ODTs (Badgujar and Mundada, 2011; Hirani et al., 2009; Yapar, 2014). Moreover, as all components are wetted and inside the bulk formulation, the prepossessing steps are much safer. Even the post-processing steps such as inspection and blistering are more sheltered in the lyophilized ODT process, as the liquid bulk formulation is usually dosed and processed directly in the primary packaging, i.e. blister. In contrast to compression, the risky direct contact of the product during and after processing is avoided in lyophilizing ODTs. Additionally, the freeze-drying technology is compatible with more complex biological active agents such as attenuated viruses (e.g. oral vaccines) and proteins (e.g. immunotherapy). On the other hand, direct compacting is the more economical method with more accessible and widely spread machinery.

The objective in this study is the characterization of the primary drying phase of a freeze-drying process in cold-formed aluminium blisters. Polyvinyl acetate-based ODT formulations were used as a product case (Vanbillemont and De Beer, 2020). The current freeze-drying literature mainly describes the case of pharmaceutical vials (Pikal, 2002). The conversion of the mechanistic description of a vial system towards more exotic blister forms is in-scope. Dynamic optimization of the primary drying phase in a freeze-drying process is shown to significantly reduce the processing times while keeping a qualitative product (De Meyer et al., 2019; Fissore et al., 2011; Vanbillemont et al., 2020c). When the critical process parameters (CPPs), i.e. shelf temperature (*T*_*s*_) and chamber pressure (*P*_*c*_), are optimized for each stage of primary drying, they can compensate for the continuously changing physical state of the product, i.e. increasing dried layer thickness and resistance. It is the aim, to describe the primary drying phase of oral lyophilizates in exotic blister forms accurately and to apply them in an mechanistically driven optimization of the process.

## 2 Materials and methods

### 2.1 Materials

Micronized acetaminophen was acquired from Mallinckrodt (Staines-upon-Thames, United Kingdom) and used as the model active pharmaceutical ingredient (API). As excipients, mannitol was used as a crystalline bulking agent from ABC Chemicals (Nazareth, Belgium), polyvinyl alcohol (PVA) 4–88 Emprove (Merck KGaA, Darmstadt, Germany) with a hydrolyzation grade of 88% as a polymeric binder and xanthan gum ACA Pharma (Waregem, Belgium) was utilized as a viscosity enhancer.

### 2.2 Methods

#### 2.2.1 Blister shape

**Fig. 1:**
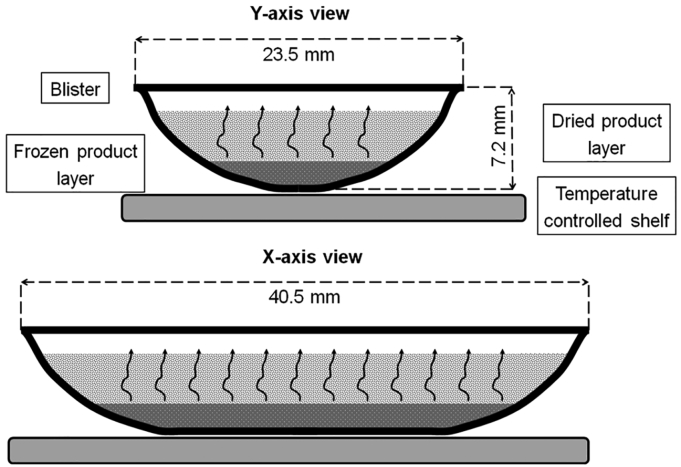
Intersection along the Y and X-axis of the blister during primary drying.

Cold-formed aluminium blisters (oPA/Alu/PVC – 25/45/60 μm) were acquired from IMA (Bologna, Italy). The blister pocket shape could be approximated by a sliced horizontal cylinder with spherical caps with a total length of 40.5 mm, chord length of 23.5 mm and height of 7.2 mm (cfr. Fig. 1). The primary drying phase of a lyophilized orally disintegrating tablet (ODT) was mechanistically modeled starting from the heat and mass balances describing the primary drying process of parenteral vials (Pikal, 2002). Several adaptations to these models were necessary to account for the acquired blister shape.

#### 2.2.2 Bulk formulation preparation

Two optimized polyvinyl acetate (PVA) based formulations from earlier research were utilized in this study as model formulations (Vanbillemont and De Beer, 2020). The exact composition of both formulations are displayed in Table 1. The high-dose formulation contained acetaminophen as a model drug substance and its composition was optimized to achieve a 500 mg dose product. Additionally, a low-dose formulation was also investigated with hydrochlorothiazide as the model. The exact concentration of all components was set gravimetrically. For PVA and Xanthan gum a dilution strategy (1 in 10 and 1 in 100, respectively) was followed to achieve an accurate and homogeneous suspension.

**Table 1:** Compositions of the high-dose acetaminophen (APAP) and low-dose hydrochlorothiazide (HCT) formulations.

Components	High-dose APAP	Low-dose HCT
	[%(w/w)]	[%(w/w)]
Drug susbtance	33.00	1.25
PVA	3.39	2.96
Mannitol	2.76	9.98
Xanthan gum	0.075	0.250
Water	ad 100	ad 100

#### 2.2.3 Heat transfer coefficient

The heat transfer from the technical fluid inside the freeze-dryer shelf to the product inside the blister was determined using a gravimetric method (Mortier et al., 2016; Pisano et al., 2011b) and was described by the heat transfer coefficient (*K*_*v*_ [W/m^2^K]). The inter-shelf distance was set to 5 cm to limit the view factor of the uncooled walls and thus the radiation effect of these warmer walls. 104 blister cups filled with 2.3 ml of deionized water were placed in a lab-scale Amsco-Finn Aqua GT4 freeze-dryer (GEA, Köln, Germany). Three thin-gauge type-K thermocouples (Labfacility, Leeds, UK) were fixed to the bottom of one edge and two center blisters (Nail et al., 2017). An edge blister was defined as one not sharing six neighboring blisters. The shelf temperature (*T*_*s*_ [K]) was brought down to −30 °C to freeze all blisters. After full solidification, the pressure was lowered to start sublimation followed by a raise of *T*_*s*_ to −15 °C and held for 2.5 h after which approximately one-third of the ice was sublimated. All blister cups were weighed before and after freeze-drying. The *K*_*v*_ experiments were repeated at different chamber pressure levels (*P*_*c*_ [Pa]) (i.e. 10, 15, 20, 25 and 30 Pa) to investigate the pressure effect. The *K*_*v*_ of all the blister cups was calculated using Eq. (1) with *m*_*sub*_ the sublimated mass [kg], Δ*H*_*sub*_ the latent heat of sublimation [J/mol], *A*_*b*_ the projected area of the blister [m^2^], *M* the molecular weight of water [kg/mol], *t*_0_ and *t*_*end*_ respectively the start and end time of primary drying [s] and *T*_*b*_ the product temperature at the bottom of the blister [K].

(1) $$K_{v}=\frac{m_{\mathrm{sub}}\Delta H_{\mathrm{sub}}}{A_{b}M\int_{t_{0}}^{t_{\mathrm{end}}}\left(T_{s}-T_{b}\right)\mathrm{dt}}$$

Δ*H*_*sub*_ of ice was estimated using an empirical relationship with the ice temperature (*T* [K]) described by coefficients *α*_*Hsub*_ till ϵ_*Hsub*_ (Mortier et al., 2015).

(2) $$\Delta H_{\mathrm{sub}}=\alpha_{H_{\mathrm{sub}}}+\beta_{H_{\mathrm{sub}}}T-\gamma_{H_{\mathrm{sub}}}T+\delta_{H_{\mathrm{sub}}}e^{-{\left(\frac{T}{\epsilon_{H_{\mathrm{sub}}}}\right)}^{2}}$$

At last, the results were split into two groups based on their location, i.e. edge and center blisters, and regressed in function of *P*_*c*_ using Eq. (3) to determine the characteristic *α* [J/m^2^sK], *β* [J/m^2^sKPa] and *γ* [1/Pa] coefficients. The inverse of the variance was used as the weights in the non-linear fitting function of Matlab 2018b (Mathworks, Natick, MA, USA).

(3) $$K_{v}=\alpha +\frac{\beta P_{c}}{1+\gamma P_{c}}$$

#### 2.2.4 Dried product mass resistance

The dried product mass resistance (*R*_*p*_ [m/s]) profile of the two polyvinyl acetate (PVA) based formulations were determined. The blister pockets were filled with 2.5 ml of each bulk suspension at a temperature of 3 °C to minimize sedimentation of the drug substance. The blisters were subsequently loaded on pre-cooled shelves (3 °C) of the Amsco-Finn Aqua GT4 freeze-dryer. Next, the blisters were freeze-dried according a conservative cycle to prevent product failures. Shelves were set to −30 °C with a cooling rate of 0.25 °C/min and attained at this setting for 30 min to solidify the products. Next, a pressure of 10 Pa was set for primary drying. Upon reaching this setting the shelf temperature was increased to −25 °C over 30 min and attained till convergence of the Pirani and Capacitance pressure sensors, indicating end of primary drying.

Two thermocouples were fitted on the bottom of random center blister cups and the average temperature was used as the bottom product temperature (*T*_*b*_ [K]). Eq. (1) was inverted to yield the sublimation rate (${\dot{m}}_{\mathrm{sub}}$ [kg/s]) from the process data. Next, the sublimated volume (*V*_*i*_ [m^3^]) at every time point (*t*_*i*_ [s]) was calculated using Eq. (4) with *t*_0_ the start of primary drying [s] and Δ*t* the time resolution [s], *θ* the porosity of the dried layer [−] and *ρ*_*ice*_ the mass density of ice [kg/m^3^]. The dried layer porosity was approached using the mass concentrations of the initial bulk suspension.

(4) $$V_{i}=\sum_{t_{0}}^{t_{i}}\frac{{\dot{m}}_{\mathrm{sub},i}\Delta t}{{\theta \rho }_{\mathrm{ice}}}$$

Subsequently, the frozen layer thickness (*L*_*ice*_ [m]) could be estimated by solving Eq. (5) with *V*_*fill*_ the filling volume [m^3^], *ρ*_*sol*_ the mass density of the bulk suspension [kg/m^3^], *R*_*b*_ the radius of the blister [m]. *R*_*b*_ was estimated using the blisters' height (*h*_*b*_ [m]) and chord length (*c*_*b*_ [m]) (inversion of Eq. (6)).

(5) $$\begin{array}{l} \frac{V_{\mathrm{fill}}\rho_{\mathrm{sol}}}{\rho_{\mathrm{ice}}}-V_{i}=\frac{\pi L_{\mathrm{ice},i}^{2}}{3}\left(3R_{b}-L_{\mathrm{ice},i}\right)\\ \;+L_{b}\left[R_{b}^{2}\arccos\left(\frac{R_{b}-L_{\mathrm{ice},i}}{R_{b}}\right)-\left(R_{b}-L_{\mathrm{ice},i}\right)\sqrt{2R_{b}L_{\mathrm{ice},i}-L_{\mathrm{ice},i}^{2}}\right] \end{array}$$

Next, the chord length (*c*_*ice*_ [m]) of the sublimation front, defined by the blister dimensions, was calculated using Eq. (6). *c*_*ice*, *i*_ was required to compute the sublimation surface (*A*_*p*_ [m^2^]) via Eq. (7) with *L*_*cyl*, *b*_ the length of the cylindrical mid-section. *L*_*cyl*, *b*_ could be estimated from the total blister length (*L*_*b*_ [m]) and chord length (*L*_*cyl*, *b*_ = *L*_*b*_ − *c*_*b*_).

(6) $$c_{\mathrm{ice},i}=2\sqrt{2R_{b}L_{\mathrm{ice},i}-L_{\mathrm{ice},i}^{2}}$$

(7) $$A_{p,i}=\pi {\left(\frac{c_{\mathrm{ice},i}}{2}\right)}^{2}+c_{\mathrm{ice},i}L_{\mathrm{cyl},b}$$

Furthermore, the sublimation interface temperature (*T*_*i*_ [K]) was estimated from the thermocouple process data (i.e. *T*_*b*_ [K]) using a Fourier conduction formula (cfr. Eq. (8)) with *λ*_*ice*_ the heat conductivity of the frozen product layer [W/mK]. The vapor pressure of water (*P*_*i*_) above the sublimation front [Pa] was estimated using an empirical relation with the sublimation temperature described by Murphy and Koop (2005) with *α*_*Pi*_ till *δ*_*Pi*_ the coefficients (cfr. Eq. (9)).

(8) $$T_{i}=T_{b}-\frac{\left(T_{s}-T_{b}\right)K_{v}L_{\mathrm{ice}}}{\lambda_{\mathrm{ice}}}$$

(9) $$P_{i}=e^{\alpha_{P_{i}}-\frac{\beta_{P_{i}}}{T_{i}}+\gamma_{P_{i}}\ln\left(T_{i}\right)-\delta_{P_{i}}T_{i}}$$

Ultimately, the dried product mass resistance (*R*_*p*_ [m/s]) could be calculated for both formulations by applying Eq. (10). The associated dried product thickness (*L*_*dr*, *i*_ [m]) was computed from the initial frozen product layer (*L*_*ice*, 0_ [m]) and *L*_*ice*, *i*_ (*L*_*dr*, *i*_ = *L*_*ice*, 0_ − *L*_*ice*, *i*_). The resultant *L*_*dr*_ versus *R*_*p*_ curves could then be regressed using Eq. (11) yielding *R*_*p*0_, *A*_*Rp*_ and *B*_*Rp*_ as coefficients.

(10) $$R_{p,i}=\frac{A_{p}\left(P_{i}-P_{c}\right)}{{\dot{m}}_{\mathrm{sub},i}}$$

(11) $$R_{p}=R_{p0}+\frac{A_{\mathrm{Rp}}L_{\mathrm{dr}}}{1+B_{\mathrm{Rp}}L_{\mathrm{dr}}}$$

#### 2.2.5 Freeze-dry microscope

The collapse temperature (*T*_*c*_) of the high-dose model formulation was investigated using a FDCS 196 freeze-dry microscopy (Linkam, Surrey, UK). The freeze-drying stage was mounted on an optical microscope (BX51, Olympus, Hamburg, Germany) and ≈ 50 μl of bulk solution was loaded on the stage. The sample was frozen to −45 °C via the Linksys32 software (Linksys 32, Linkam, Surrey, UK) and pressure equilibrated at 1 Pa using a rotary vane vacuum pump (E2M1.5, Edwards, Nazareth, Belgium). The product temperature was increased in steps of 2 °C with an equilibration period of 5 min. Digital images were taken at the end of the equilibration period and overlaid. The product temperature where slight alterations to the dried product structure were noticeable, were interpreted as the collapse temperature (*T*_*c*_ [K]).

#### 2.2.6 Primary drying optimization

An optimal primary drying profile was computed by employing the determined model parameters (i.e. *K*_*v*_ and *R*_*p*_ coefficients) as described in the previous sections. For the high-dose formulation a 1.52 ml tablet in the above described blister system was modeled to yield a ODT with 500 mg dose of acetaminophen. The optimization exercise started at a *T*_*s*_ of −30 °C and a constant *P*_*c*_ of 10 Pa. For every prediction interval (i.e. 30 s), a machine capability interval was constructed for *T*_*s*_ which was dependent on the previous *T*_*s*_ setpoint and the maximal cooling/heating rate (i.e. 1.4 °C/min) with a resolution of 0.1 °C. Every point in the machine capability interval was evaluated by solving Eqs. (2), (9), (12), (13) simultaneously for *T*_*i*_. A *T*_*s*_ resulting in a *T*_*i*_ above *T*_*c*_ was invalidated and the setting with the highest sublimation speed was selected as the operating condition. This method resulted in a dynamic optimization of *T*_*s*_.

Eq. (12) is achieved by combining Eq. (1), (10) and solving it for *P*_*i*_. It describes a planar sublimation interface that moves from top to the bottom of the unit dose while forming a dried product layer above it. Next, Eq. (13) describes the temperature difference over the frozen product layer. This system of equations is already thoroughly described in literature (De Meyer et al., 2017; Leys et al., 2020; Mortier et al., 2015; Vanbillemont et al., 2020c).

(12) $$P_{i}=-\frac{-A_{p}\Delta H_{\mathrm{sub}}P_{c}-A_{b}K_{v}R_{p}{\mathrm{MT}}_{s}+A_{b}K_{v}R_{p}{\mathrm{MT}}_{i}+A_{b}K_{v}R_{p}M\Delta T}{A_{p}\Delta H_{\mathrm{sub}}}$$

(13) $$\Delta T=\frac{a\frac{\left(L_{\mathrm{tot}}-L_{\mathrm{dr}}\right)\left(P_{i}-P_{c}\right)}{R_{p}}-b\left(L_{\mathrm{tot}}-L_{\mathrm{dr}}\right)\left(T_{s}-T_{i}\right)}{1-b\left(L_{\mathrm{tot}}-L_{\mathrm{dr}}\right)}$$

For the purpose of the optimization only the edge blister population, i.e. the edge *K*_*v*_ parameters, were in scope for determining the *T*_*s*_. However, the center blister population, i.e. the center *K*_*v*_ parameters, were utilized to determine the total length of primary drying phase as these blister dry slighter slower due to a lower heat transfer. It has to be stressed that the optimized heating strategy will only be valid for the proposed pressure level.

#### 2.2.7 Primary drying verification

The optimal settings calculated according section 2.2.6 were verified by filling 1.52 ml of the high-dose formulation in the blister cups and loading them on 3 °C pre-cooled shelves. Next, the liquid was solidified by lowering the shelves at 0.25 °C/min towards −30 °C after which the pressure was lowered to 10 Pa. Upon reaching the pressure setpoint, the dynamic *T*_*s*_ trajectory was loaded and executed with a setpoint resolution of 30 s while maintaining the chamber pressure by capacitance sensor (*P*_*c*, *Cap*_ [Pa]) at 10 Pa. The last setpoint was prolonged for 1 h to ensure all products were dry of ice before starting secondary drying. For secondary drying, a 1 °C/min ramp towards 25 °C was implemented and maintained for 1 h. The primary drying phase was monitored by inserting a type-K thermocouple in an edge and center population blister and the chamber pressure by Pirani guage (*P*_*c*, *Pir*_ [Pa]) was also recorded. The endpoint of primary drying was evaluated by the differential pressure measurement of both pressure gauges (*P*_*ratio*_ [−]) (Vanbillemont et al., 2020c). All parameters values are listed in Table 2.

**Table 2:** Parameters list used in the regression of the freeze-drying parameters and simulation of the primary drying phase.

Description	Symbol	Value	Unit
Time resolution simulation	Δ*t*	60	s
Porosity (low−/high-dose)	*θ*	0.8556 / 0.6079	(−)
Mass density ice	*ρ*_*ice*_	918	kg/m^3^
Mass density bulk	*ρ*_*sol*_	1018	kg/m^3^
Heat conductivity frozen product	*λ*_*ice*_	2.3	W/mK
*P*_*i*_ coefficient	*α*_*Pi*_	9.550426	Pa
*P*_*i*_ coefficient	*β*_*Pi*_	5723.2658	K
*P*_*i*_ coefficient	*γ*_*Pi*_	3.53068	1/K
*P*_*i*_ coefficient	*δ*_*Pi*_	0.00728332	Pa
Δ*H*_*sub*_ coefficient	*α*_*H*_*sub*__	4.68 × 10^4^	J/mol
Δ*H*_*sub*_ coefficient	*β*_*H*_*sub*__	35.9	J/molK
Δ*H*_*sub*_ coefficient	*γ*_*H*_*sub*__	0.0741	J/molK^2^
Δ*H*_*sub*_ coefficient	*δ*_*H*_*sub*__	542	J/mol
Δ*H*_*sub*_ coefficient	*ε*_*H*_*sub*__	124	K²
Conversion factor a	*a*	889,200	–
Conversion factor b	*b*	1.02	–
Molecular weight water	*M*	18.01528 × 10^−3^	kg/mol
Chord of blister	*c*_*b*_	23.5 × 10^−3^	m
Height of blister	*h*_*b*_	7.2 × 10^−3^	m
Total length of blister	*L*_*b*_	40.5 × 10^−3^	m
Filling volume	*V*_*fill*_	2.50 / 1.52 × 10^−6^	m³

## 3 Results and discussion

### 3.1 Primary-drying modeling

Cold-formed blisters typically have rounded edges with a long radius to prevent excessive strain on the blister material when cold-pressing the shape. Sharp angles in the shape would otherwise result in a rupture of the blister foil. However, this will lead to more exotic product shapes than the cylinders who are typically described in parenteral freeze-drying (Pikal, 2002; Vanbillemont et al., 2020b). A mechanistic description of the product shape using a sliced horizontal cylinder with spherical caps model was proposed and implemented in the heat and mass balances of the primary drying phase (i.e. Eq. (5)). Next, essential freeze-drying parameters (*K*_*v*_ and *R*_*p*_) were determined by using two PVA-based ODT formulations as a model. The impact of a formulation on the freeze-drying process is quite significant as it determines the characteristic *R*_*p*_ versus *L*_*dr*_ profile. It is therefore expected that both formulations will show different drying dynamics. A separate process optimization for each formulation would be required. In this work, the focus of optimization is placed on the primary drying phase where the highest efficiency gain is typically possible (Fissore et al., 2011).

#### 3.1.1 Heat transfer coefficient

**Fig. 2:**
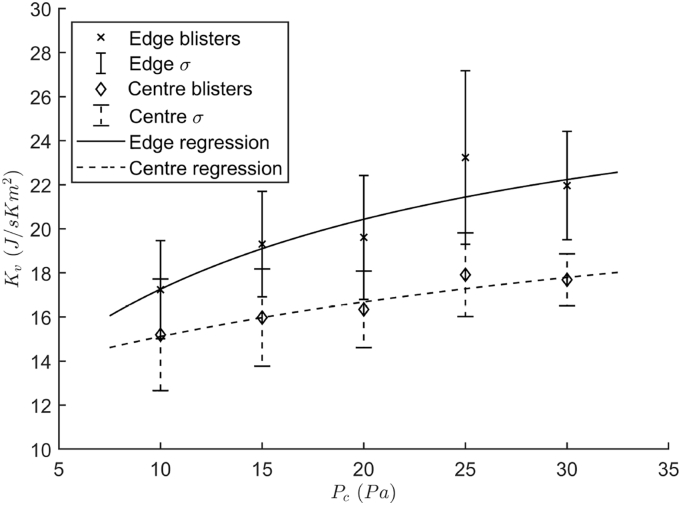
Heat transfer coefficient of a cold-form aluminium blister in function of chamber pressure. Solid lines and crosses represent the edge population whereas dashed lines and diamonds represent the center population. Errorbars depict the experimental standard deviation.

Gravimetric heat transfer experiments were executed with cold-form aluminium blisters at five pressure levels (cfr. Fig. 2). The inverse of the variance of the data points was used as the weights. *K*_*v*_ coefficients were fitted for both the edge and center blister population. The *K*_*v*_ of the edge blisters could be described between 10 and 30 Pa with an *α* of 10.1917 J/m^2^sK; a *β* of 1.1408 J/m^2^sKPa and a *γ* of 0.0614 1/Pa. Contrarily, the center population yielded an *α* of 12.7208 J/m^2^sK, a *β* of 0.3015 J/m^2^sKPa and a *γ* of 0.0261 1/Pa.

When comparing the *K*_*v*_ of blisters with these of pharmaceutical vials described by Vanbillemont et al. (2020c), determined using a similar protocol and freeze-dryer, the *K*_*v*_ increase of blisters with the chamber pressure (*P*_*c*_) was rather limited. Blisters showed a maximal gain of 25% and 15% for the edge and center group respectively, compared to the doubling of the *K*_*v*_ for pharmaceutical vials in the 10 to 25 Pa interval. Due to the horizontal cylindrical shaped blister, only a very limited direct contact area was available for conduction. Moreover, the limited interaction of *K*_*v*_ with *P*_*c*_ suggests that there was only a minor contribution of the convection term (Pisano et al., 2011a). Hence, it should be concluded that the energy transfer was mostly made through radiation. Notwithstanding, the relative difference between the edge and center population was quite similar for blisters (17.6%) in comparison with the vials (20.8%). It has to be noted that the inter-shelf distance was four times smaller for the blister compared to the vials. Larger inter-shelf distances would probably aggravate the edge-center inhomogeneity, as a higher view factor for the uncooled walls would be present. Nonetheless, the blisters displayed a huge spread in *K*_*v*_ across both the edge and center populations. Due to the nature and shape of the blisters, the contact area with the shelves was very variable, presumably leading to different amounts of heat transfer by conduction and convection. This large *K*_*v*_ variation will lead to considerable intra-batch inhomogeneity, characteristic to batch freeze-drying, as some blisters will dry faster and are more at risk for collapse or meltback (Pisano et al., 2011a, Pisano et al., 2020).

#### 3.1.2 Dried product mass resistance

**Fig. 3:**
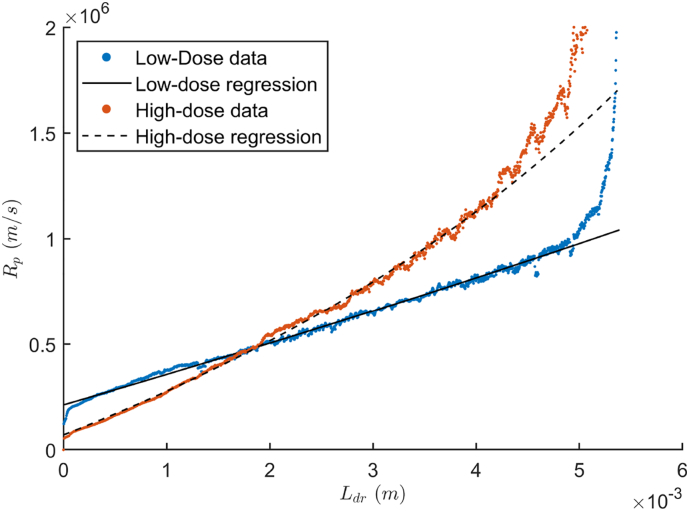
The dried product mass resistance in function of the dried product layer thickness for both the low-dose and high-dose formulations with the regressions as line plots.

**Fig. 4:**
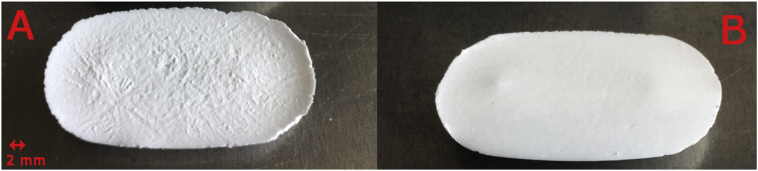
Picture of the (A) optimized low-dose and (B) high-dose ODT. A skin effect is visible on the low-dose tablet.

Both the optimal low- and high-dose formulation were monitored during primary drying using thermocouples, enabling the estimation of the dried product mass resistance (*R*_*p*_) and the progress of drying by *L*_*dr*_. Fig. 3 illustrates the *R*_*p*_ versus *L*_*dr*_ trajectories. Both formulations showed a near-linear increase of *R*_*p*_ with *L*_*dr*_, which is typically reported for mannitol based formulations (Kuu et al., 1995, Kuu et al., 2006). The high-dose acetaminophen formulation showed a steeper increase but started lower compared to the low-dose HCT ODT. The steeper increase of the acetaminophen ODT was probably due to the lower porosity. Porosity influences next to the tortuosity, the effective diffusivity of a porous network (Pisano et al., 2017). The higher initial *R*_*p*_ of the HCT formulations could most likely be attributed to the formation of a skin on top of the tablet, not observed with the acetaminophen ODTs (cfr. Fig. 4). Skin formation is typically due to the concentration of solutes at the top of the cake during the freezing or annealing phase (Esfandiary et al., 2016). Further optimization of the freezing phase or the effect of annealing should be investigated, to examine if more elegant and faster-drying HCT ODTs could be manufactured. As binary-polymer systems are often responsible for inducing phase-separation during freezing, lowering the xanthan gum concentration could perhaps solve the skin-effect (Izutsu et al., 1996; Padilla et al., 2011). However, this approach can only be investigated with an HCT-grade displaying a smaller particle size, lowering the risk of sedimentation (Vanbillemont and De Beer, 2020).

A regression of the *R*_*p*_ versus *L*_*dr*_ trajectories was made and is depicted in Fig. 3 by a solid line for HCT and dashed line for acetaminophen. Only the first 4.5 mm of the trajectories were used since *R*_*p*_ values at higher *L*_*dr*_ displayed an exponential increase. These deviations at the end of primary drying are already extensively reported in the literature and are caused by an upshift of the thermocouple values due to partial completion of sublimation inside one unit-dose (Kuu et al., 2006; Van Bockstal et al., 2018; Vanbillemont et al., 2020c). These deviations could even be exaggerated by the cylindrical shape of the blister resulting in a rapidly decreasing product surface at the end of primary drying. The low-dose formulation regression yielded three significant coefficients (*R*_*p*0_: 2.12 × 10^5^ m/s; *A*_*Rp*_: 1.42 × 10^8^ 1/s and *B*_*Rp*_: −14.39 1/m) with a RSME of 1.67 × 10^4^ m/s. Similarly, the regression of the optimal high-dose formulation resulted in three significant coefficients (*R*_*p*0_: 7.05 × 10^4^ m/s; *A*_*Rp*_: 1.92 × 10^8^ 1/s and *B*_*Rp*_: −68.30 1/m) with a RSME of 1.89 × 10^4^ m/s.

#### 3.1.3 Collapse temperature

**Fig. 5:**
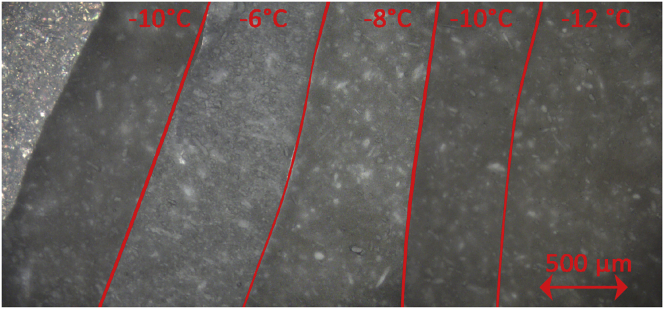
Overlay of images of the freeze-dry microscope of the high-dose formulation with red line the border of the overlays. Ice is still present at the left of the image. (For interpretation of the references to color in this figure legend, the reader is referred to the web version of this article.)

Fig. 5 displays the microstructure near the sublimation interface at different product temperatures. The product temperature was increased in steps of 2 °C from right to left (−12 till −6 °C) until a clear structural difference appeared. Finally, the last safe operating temperature was re-evaluated again (−10 °C). The frozen product layer is still visible in the top left corner of Fig. 5. As evidenced by the color saturation of the dried layer segments, product temperatures of −10 °C or less did not cause alterations to the structure of the dried layer. However, when a product temperature of −8 °C was set, a slight change in color was perceivable, and upon setting −6 °C lower level acetaminophen crystals became more visible. It has to be noted that due to the higher viscosity of the high-dose formulation, a larger sample size than usual was loaded on the freeze-dry microscope. This could have influenced the accuracy of the method and clarity of the images. Single individual pores and fissures could not be distinguished on the highest magnification due the thicker then usual sample size. However, the saturation change and appearance of more acetaminophen (i.e. white) crystals of the underlying layers is most probably due to micro-collapse of the dried layer. Product temperatures of −10 °C or below are hence assumed to be safe for the cause of process optimization.

#### 3.1.4 Primary drying simulation

The collapse temperature (*T*_*c*_) is a very critical parameter in a primary drying process design. If the product temperature surpasses this limit, it is at a big risk of collapse. Collapse could negatively influence the product quality as disintegration time could be lengthened and possibly impacting the mechanical strength of the tablet (Rhys, 2013). In this study, *T*_*c*_ of the high-dose bulk suspensions was measured via FDM. The *T*_*c*_ can be considered as the upper limit for *T*_*i*_ during primary drying modeling (Mortier et al., 2015; Van Bockstal et al., 2017).

**Fig. 6:**
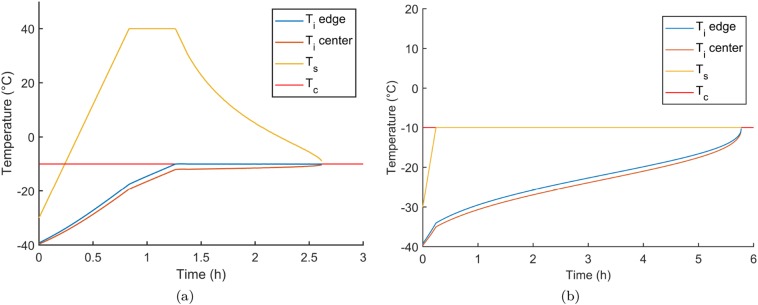
Process data predictions of the (a) optimized primary drying trajectory for the high-dose product and (b) traditional cycle.

A dynamic setting strategy for *T*_*s*_ was applied to maximize the sublimation rate while keeping the product safe (*T*_*i*_ < *T*_*c*_). It appears in Fig. 6(a) that an aggressive start of primary drying was possible for the high-dose formulation. Such an aggressive start will allow for a significant reduction in processing time compared to a traditional cycle (cfr. Fig. 6(b)). Next, the upper machine limit of 40 °C is reached and the *T*_*s*_ is attained at this limit till the *R*_*p*_ starts to be limiting towards the *T*_*i*_. After 1.5 h the dynamic optimization exercise reduces the *T*_*s*_ gradually because of the increasing *R*_*p*_ and lowering of the *A*_*p*_ with the progress of primary drying. However, it should be noted that quite significant changes in *T*_*s*_ are required to optimize the process in comparison with batch processes (Vanbillemont et al., 2020c). The main reason for this is the lower *K*_*v*_ parameter of blisters, which require a bigger temperature differential between shelf and product to achieve similar heat fluxes. From Fig. 6(a) it is clear that in the first half of the process, the machine limits in maximal heating power and the upper absolute temperature prevents even more aggressive drying profiles since the product temperatures are still far below *T*_*c*_. Using a dynamic strategy to dramatically reduce process time would even be more advantageous in continuous manufacturing concepts with a more direct heat transfer. Concepts such as the ones proposed by Corver (2012) and Pisano et al. (2019) but adapted to ODTs would eliminate these rather conservative machine limits as the process is split in the spatial dimension rather then time. Moreover, such concepts for ODTs could eventually eliminate the dramatic heat transfer variation observed in batch processing of blisters (Capozzi et al., 2019; Lammens et al., 2018). Due to the relative high *T*_*c*_, a quite aggressive drying protocol could be optimized which results in a nominal primary drying time of 2.62 h. When considering a traditional protocol (cfr. Fig. 6(b)) with a maximum *T*_*s*_ of −10 °C, a total primary drying time of 5.77 h was predicted. Applying a dynamic heating protocol can shorten the lengthy primary drying time with 55%.

#### 3.1.5 Primary drying verification

**Fig. 7:**
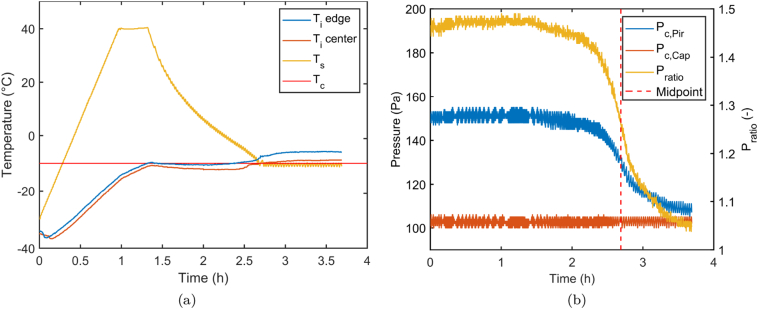
Process data from the primary drying verification run of the high-dose formulation. (a) Temperatures plot and (b) the pressures plot where the red dashed lines represents the midpoint of the end of primary drying by comparative pressure measurement. (For interpretation of the references to color in this figure legend, the reader is referred to the web version of this article.)

Fig. 7 depicts the process data of the verification run of the optimized process trajectory (cfr. Section 3.1.4). Both thermocouple values were updated using Eq. (8) to get an estimate of the *T*_*i*_ of the center and edge blister population (red and blue curve in Fig. 7(a), respectively). These sublimation interface temperatures show an identical shape as predicted in Fig. 6. Moreover, the ice sublimation interface temperature of the edge vials is situated around −10 °C, between 1.5 and 2.5 h as instructed by the mechanistic model optimization. Both observations indicates accurate model predictions. When looking closer to the endpoint of primary drying, thermocouple values start deviating from their trajectory at 2.53 and 2.70 h, indicating a loss of contact with ice. These values only deviate 3% from the model prediction of 2.62 h. Furthermore, the midpoint of the comparative pressure measurement is located at 2.69 h (cfr. Fig. 7(b)) (Patel et al., 2010). However, due to intra-batch variations, and more specifically the relative big variability of *K*_*v*_, the end of primary drying for all unit doses was only observed at 3.68 h. A optimization methodology that encompasses all sources of variability would therefore be more appropriate as it could lead to a mathematical estimation of the risks on process failures. Incorporation of the proposed mechanistical model describing cold-forms blisters into the model-based optimization and control strategy proposed by Vanbillemont et al. (2020c) would be an opportunity for further research to solve this problem. Several tablets were unblistered and no signs of collapse or meltback were visible. Moreover, the tablets showed very similar product characteristics as described in Vanbillemont and De Beer (2020) with a disintegration time of around 7 s and a mechanical strength around 3 MPa (Vanbillemont et al., 2020a).

## 4 Conclusion

A mechanistic model of the primary drying phase of oral lyophilizates was proposed which could accurately describe the changing product shape due to the use of cold-form blisters. Furthermore, the heat transfer of blisters was characterized in a lab-scale freeze-dryer. It was found that the heat transfer showed a high blister-to-blister variability and was only slightly influenced by the chamber pressure. Next, the dried layer mass resistance was computed for two PVA-based formulations. The mechanistic model could be utilized for a dynamic optimization of the primary drying phase resulting in ultra-short primary drying times (3.68 h) for a 500 mg acetaminophen ODT formulation.

## Declaration of Competing Interest

The authors declare that they have no known competing financial interests.
